# Addressing Unintentional Exclusion of Vulnerable and Mobile Households in Traditional Surveys in Kathmandu, Dhaka, and Hanoi: a Mixed-Methods Feasibility Study

**DOI:** 10.1007/s11524-020-00485-z

**Published:** 2020-10-27

**Authors:** Dana R. Thomson, Radheshyam Bhattarai, Sudeepa Khanal, Shraddha Manandhar, Rajeev Dhungel, Subash Gajurel, Joseph Paul Hicks, Duong Minh Duc, Junnatul Ferdoush, Tarana Ferdous, Nushrat Jahan Urmy, Riffat Ara Shawon, Khuong Quynh Long, Ak Narayan Poudel, Chris Cartwright, Hilary Wallace, Tim Ensor, Sushil Baral, Saidur Mashreky, Rumana Huque, Hoang Van Minh, Helen Elsey

**Affiliations:** 1 Department of Demography and Social Statistics, University of Southampton, Highfield Campus Building 58, Southampton, SO17 1BJ UK; 2 Health Research and Social Development Forum-International, Kathmandu, Nepal; 3 Nuffield Centre for International Health and Development, University of Leeds, Leeds, UK; 4 Hanoi University of Public Health, Hanoi, Vietnam; 5 Centre for Injury Prevention and Research – Bangladesh, Dhaka, Bangladesh; 6 Advancement through Research and Knowledge Foundation, Dhaka, Bangladesh; 7 School of Medicine, The University of Notre Dame Australia, Fremantle, WA Australia

**Keywords:** Nepal, Vietnam, Bangladesh, Gridded population sampling, GridSample, OpenStreetMap, GeoODK, Cross-sectional design, Urban, Household survey

## Abstract

The methods used in low- and middle-income countries’ (LMICs) household surveys have not changed in four decades; however, LMIC societies have changed substantially and now face unprecedented rates of urbanization and urbanization of poverty. This mismatch may result in unintentional exclusion of vulnerable and mobile urban populations. We compare three survey method innovations with standard survey methods in Kathmandu, Dhaka, and Hanoi and summarize feasibility of our innovative methods in terms of time, cost, skill requirements, and experiences. We used descriptive statistics and regression techniques to compare respondent characteristics in samples drawn with innovative versus standard survey designs and household definitions, adjusting for sample probability weights and clustering. Feasibility of innovative methods was evaluated using a thematic framework analysis of focus group discussions with survey field staff, and via survey planner budgets. We found that a common household definition excluded single adults (46.9%) and migrant-headed households (6.7%), as well as non-married (8.5%), unemployed (10.5%), disabled (9.3%), and studying adults (14.3%). Further, standard two-stage sampling resulted in fewer single adult and non-family households than an innovative area-microcensus design; however, two-stage sampling resulted in more tent and shack dwellers. Our survey innovations provided good value for money, and field staff experiences were neutral or positive. Staff recommended streamlining field tools and pairing technical and survey content experts during fieldwork. This evidence of exclusion of vulnerable and mobile urban populations in LMIC household surveys is deeply concerning and underscores the need to modernize survey methods and practices.

**Electronic supplementary material:**

The online version of this article (10.1007/s11524-020-00485-z) contains supplementary material, which is available to authorized users.

## Introduction

In low- and middle-income countries (LMICs), household survey methods have remained consistent, while population trends have changed substantially over 40 years. This mismatch has likely increased exclusion of vulnerable and mobile populations from survey data. LMIC survey best practices were established when LMICs were majority rural by agencies that have been critiqued for holding a “sedentary bias” in development initiatives [1, 2]. Globally, human mobility has increased substantially over the last two decades, and today, most LMICs are in the midst of urban transitions, or will be soon [3]. An estimated 2.5 billion people will be added to the planet by 2050, with 90% of that population increase concentrated in Asian and African cities alone [4]. While rates of urban growth in LMIC cities are consistent with rates previously observed in high-income countries, the number of people added to LMIC cities today creates unprecedented scenarios of urbanization. For example, Lagos Nigeria, Delhi India, and Dhaka Bangladesh are each expected to add more than 700,000 people per year through 2030 [4].

Rapid in-migration to LMIC cities is accompanied by increased socioeconomic inequalities, growth in slum populations, and housing crises, all of which contribute to increasingly complex living arrangements [5, 6]. As urbanization changes the structure and nature of communities and households in LMICs [7], survey methods must evolve in response. To date, most surveys about slum communities are conducted as one-off exercises and focus on a selection of slums in a city [8, 9]. A few national surveys have explicitly sampled and reported about slum dwellers in all urban areas (e.g., the 2013 Bangladesh Urban Health Survey [10]) or select cities (e.g., 2015–16 India National Family Health Survey [11] in eight cities).

The largest survey programs in LMICs include the Demographic and Health Surveys (DHS), Multiple Indicator Cluster Surveys (MICS), and Living Standard Measurement Surveys (LSMS), which essentially use the same methods and tools [12]. Collectively, these programs have performed nearly 700 national surveys in more than 130 countries since 1980. Across these surveys, census enumeration areas (EAs) are sampled with probability proportional to population size (PPS), households in selected EAs (i.e., clusters, primary sampling units) are mapped and listed, approximately 20 households are sampled in each cluster, and interviewers return later to administer questionnaires to selected households [13–15]. Among DHS surveys conducted since 2000, the average sample frame was 7 years old (up to 30 years old), and 94% of surveys used the previous census as a sample frame, while the remaining 6% used an official list of areas or households [16]. By relying on census sample frames, unregistered and special populations excluded from the standard census are intentionally omitted from surveys including the homeless, internally displaced people, refugees, informal slum dwellers, nomadic populations, and institutional populations [6, 17].

Unintentional exclusion of vulnerable and mobile populations, particularly slum dwellers, can additionally occur in three ways. First, if structures built and occupied since the last census are over-represented in deprived areas, vulnerable and mobile populations are systematically under-represented in the first-stage sample frame. Second, two-stage sample designs result in a gap of several months between the mapping-listing and interview activities, resulting in systematic non-response from vulnerable and mobile populations not present at time of interview, and exclusion of recently occupied dwellings (living spaces). Third, disproportionate exclusion of vulnerable and mobile populations can result from poorly defined or difficult to operationalize mapping-listing protocols in the time allotted for fieldwork, for example, assuming that one household occupies each dwelling. In this case, systematic under-listing of vulnerable and mobile households who share a dwelling results in their exclusion during the second stage of sampling [18].

These three issues are labeled coverage error, non-response error, and sampling error, respectively, in the total survey error framework, and threaten to bias survey results [19]. Additional measures of survey data relevance are of concern. Given the use of survey results by decision-makers to make inferences about the general population, intentional omission of the homeless, displaced populations, informal settlers, and others due to use of census sample frames threatens relevance of survey results, particularly with respect to social and economic indicators [19]. Furthermore, without maps of deprived/non-deprived urban areas [20], the survey results of the urban poorest are masked, or hidden, in aggregated urban averages resulting in limited relevance of survey results for decision-making [19].

In recent years, national surveys that developed field-referenced slum/non-slum urban sample frames in Bangladesh [10] and India [11] found stark inequalities in health outcomes, access to health care, living conditions, and livelihood opportunities between slum and non-slum residents. A comparison of stratified slum/non-slum surveys with routine national surveys in Bangladesh, India, Kenya, and Egypt, points to conditions of the urban poorest being masked in urban averages, under-sampling of slum populations in non-stratified urban samples, or both [21]. These analyses follow years of work to highlight the absence of data about the urban poorest in censuses and surveys [8, 22]. While there are multiple other sources of slum population data in select communities, districts, or cities from single cross-sectional surveys [9–11], qualitative studies [23], community-based initiatives [24], and the INDEPTH longitudinal Demographic and Health Surveillance System [25], representative and routine measurement of populations in slums and other deprived areas via national surveys has yet to be achieved [20]. Crucially, national surveys are used to measure progress against one-fourth of the Sustainable Development Goal (SDG) indicators [26]. If current survey methods systematically under-represent and mask vulnerable and mobile urban populations, our understanding of progress toward the SDGs is fundamentally flawed.

To address problems of unintentional exclusion of vulnerable and mobile households in surveys, the Surveys for Urban Equity (SUE) project piloted and evaluated three survey innovations in Kathmandu, Dhaka, and Hanoi: (1) use of modeled gridded population data as a sample frame which was assumed to be more current and have better coverage of the entire population than census, (2) area-microcensus sample design to remove the time-lag between mapping-listing and interviewing, and (3) mapper-lister protocols including a script, OpenStreetMap and OpenDataKit tools, and a broadened household definition to identify atypical dwellings and households. We were not able to obtain maps of deprived/non-deprived areas to stratify the surveys to address problems of robustness. Here, we present results of the pilot including the extent to which populations were unintentionally excluded from a standard survey design. Further, we evaluate the feasibility, cost, and skills required to implement our novel methods in complex urban settings.

## Methods

We evaluated whether three survey innovations resulted in samples of different types of households and individuals compared with standard surveys. To establish feasibility of the innovations, we recorded costs and team skills required, and conducted focus group discussions (FGDs) to explore enumerator experiences.

### Setting

We selected Kathmandu, Nepal; Dhaka, Bangladesh; and Hanoi, Vietnam, as they typify different points on the urbanization trajectory. The pace of growth in South Asia has particularly strained urban housing markets, increasing the number of people living in atypical arrangements and locations [3]. While some poorer households live in informal settlements, others live in economically heterogeneous neighborhoods [3]. In Kathmandu and Dhaka, for example, it is common for the building owner to occupy the top floor, rent the middle floor to a middle-class family, and rent the bottom floor to multiple low-wage workers. In Vietnam, old, cramped buildings continue to house the economically and socially vulnerable, while migrant laborers live in multiple-occupancy inadequate structures near work [27]. We sampled the entire Kathmandu Valley and purposefully chose to survey a slum and an economically mixed ward in Dhaka and an economically mixed district with a large migrant population in Hanoi. The Hanoi survey occurred soon after a government campaign to evict illegal occupants.

### Study Design and Protocol

In 2017 and 2018, we conducted three cross-sectional household surveys in Kathmandu, Dhaka, and Vietnam [28].

#### Coverage Area

**Fig. 1:**
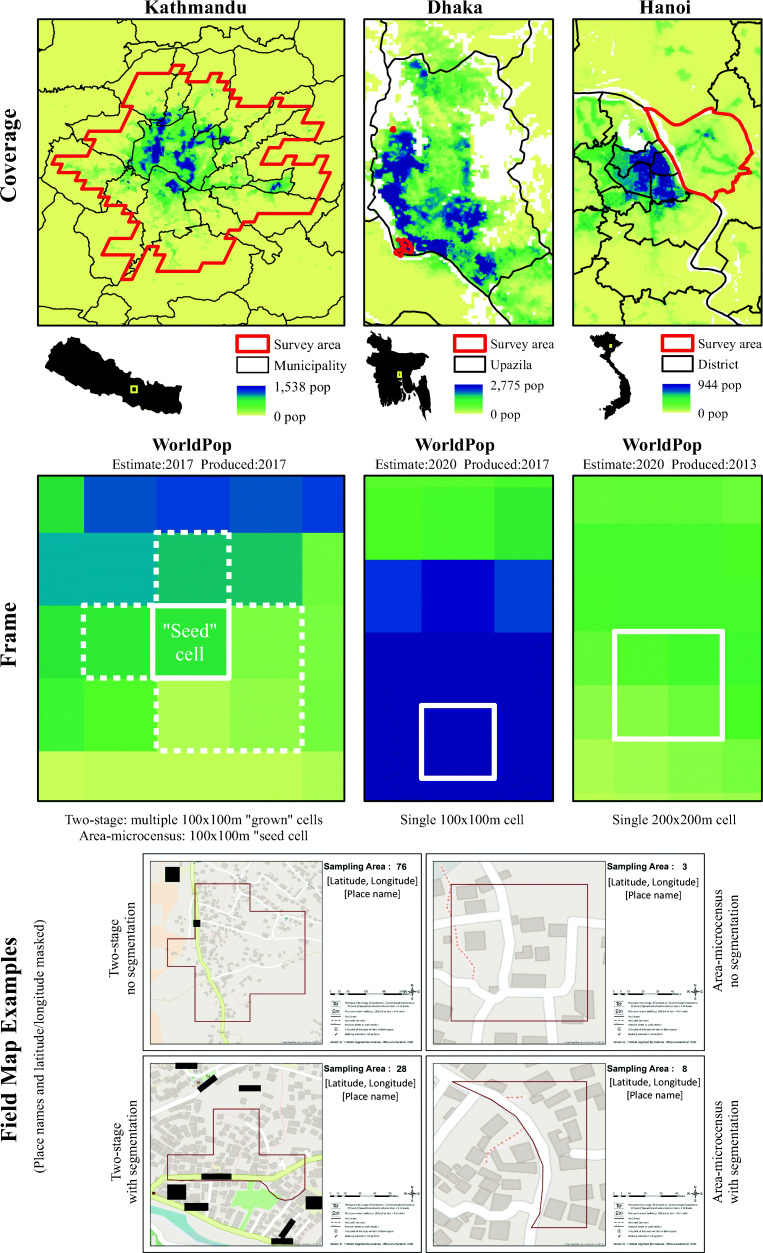
Surveys for Urban Equity coverage area boundaries, gridded population sample frames, and example field maps in Kathmandu, Dhaka, and Hanoi

The survey in Kathmandu was of the general population, while the surveys in Dhaka and Hanoi focused in areas where vulnerable and mobile population were likely located. Nepal’s government is in transition to a new federal republic system, and administrative boundaries were recently updated. Old Kathmandu municipality boundaries only included the city center, while new municipality boundaries included rural communities beyond the peri-urban reach [29]. To ensure coverage of the functional city, we used the Global Human Settlement (GHS) layer of 1 × 1 km grid cells defining “high dense urban” areas (Fig. 1). In Dhaka, the survey covered one ward and one slum community, and in Hanoi, the survey covered one district (Fig. 1).

#### Sample Size

A cluster sample of 20 households was chosen for ease of fieldwork, and to be consistent with other routine surveys such as the DHS, MICS, and LSMS. The survey in Kathmandu targeted 1200 households in 60 clusters to estimate depression and injury prevalence with a maximum 95% confidence interval of ± 4.27% (assuming the most conservative scenario where an indicator is estimated at 50%) [28]. This assumes a design effect of 1.41 (the mean design effect across all indicators for men and women in urban Nepal in the 2011 DHS) [30], a household and an individual response rate of 0.98 and 0.93, respectively, and one eligible individual per household. The Dhaka and Hanoi surveys targeted 400 households in 20 clusters each, with dual aims of evaluating transferability of SUE innovations while providing sufficient sample size to estimate key demographic and poverty indicators ± 5% with 95% confidence for indicators estimated at 50%.

#### Back-up Clusters

Given the chance of selecting areas without residential buildings (e.g., airport or factory buildings) from gridded population data and the possibility of selecting cells with no buildings, we selected 30% back-up clusters for each sample. This meant that we sampled 78 clusters in Nepal, and 26 clusters in Dhaka and Hanoi, before randomly assigning 60 (or 20) clusters to the main sample. If a sampled cluster had no residential buildings, then it was replaced with a randomly selected back-up cluster. Four additional back-up clusters were sampled in Hanoi after masking already selected clusters, because more than 6 clusters were dropped.

#### Sample Design

Area-microcensus sampling (akin to compact segment sampling [31, 32]) means that all households in a cluster are sampled, allowing the household listing and interviews to occur on the same day. Area-microcensus sampling also allows inclusion of populations typically omitted from surveys by design. In concept, area-microcensuses can be performed in clusters of any size, though in practice, smaller clusters are preferred to reduce inter-cluster correlation [33]. Furthermore, area-microcensus sampling can be performed after multiple stages of sampling, which is a common practice in surveys that use a gridded population sample frame [33]. In this study, all area-microcensuses occurred after a single stage of sampling. In Kathmandu, we randomized half of the clusters to an area-microcensus arm and the other half to a two-stage arm to compare survey designs, and treated the arms as strata (Table 1). In Dhaka, we used an area-microcensus design, stratified by ward/community with proportional allocation. The Hanoi survey followed an area-microcensus design and was not stratified.

**Table 1:** Number of clusters and households (unweighted), sample weights, and design effects by survey.

	Kathmandu				Dhaka		Hanoi	
Design								
Coverage	Kathmandu Valley		Kathmandu Valley		One ward + one slum		One district	
Stages and stratification	Two-stage, stratified by stage		Area-microcensus, stratified by stage		Area-microcensus, stratified by area		Area-microcensus, no strata	
Sample Frame	WorldPop 2017 (est. produced 2017)		WorldPop 2017 (est. produced 2017)		WorldPop 2020 (est. produced 2017)		WorldPop 2020 (est. produced 2013)	
Clusters								
Cluster definition	Multiple 100 × 100 m cells, ~ 200 households		Single 100 × 100 cell, ~ 20 households		Single 100 × 100 cell, ~ 20 households		Single 200 × 200 cell, ~ 20 households	
Targeted	30		30		20		20	
Dropped and replaced	6		3		0		9	
Sampled	30		30		20		20	
Segmented	15		7		20		18	
Households								
Proportion sampled	~ 10%		100%		100%		100%	
Targeted	600		600		400		400	
Sampled—SUE	581		599		382		463	
Sampled—DHS/MICS(% of SUE definition)	578 (99%)		538 (90%)		318 (83%)		412 (89%)	
Sampled—LSMS (% ofSUE definition)	578 (99%)		538 (90%)		343 (90%)		434 (94%)	
Household response rate	581/600 (96.8%)		599/678 (88.3%)		382/387 (98.7%)		463/560 (82.7%)	
SUE sample weights - Mean (range)	1.673 (0.298–5.524)		0.347 (0.157–0.985)		1.016 (0.113–2.595)		1.005 (0.196–4.123)	
DHS/MICS sample weights - Mean (range)	1.581 (0.300–5.283)		0.346 (0.152–0.953)		1.012 (0.107–2.604)		0.931 (0.196–4.123)	
Field work								
Mapping-listing	Households		Dwellings, then households (by interviewer)		Dwellings, then households (by interviewer)		Dwellings, then households (by interviewer)	
Interviewing	Weeks after mapping-listing		Same day a household listing		Same day a household listing		Same day a household listing	
Design effects (SUE)	Mean/prop. (SE)	DEFT	Mean/prop. (SE)	DEFT	Mean/prop. (SE)	DEFT	Mean/prop. (SE)	DEFT
HH size	3.9 (0.111)	1.53	3.4 (0.137)	1.97	4.2 (0.178)	1.87	3.662 (0.110)	1.34
HHs per dwelling	1.0 (0.011)	2.11	1.9 (0.433)	4.20	2.2 (0.189)	2.68	Not recorded	–
HHs per PSU	19.5 (0.173)	4.42	24.9 (2.691)	5.40	20.9 (1.588)	4.96	34.6 (3.756)	6.05
Residential building	0.734 (0.023)	1.27	0.682 (0.075)	3.95	0.738 (0.065)	2.89	0.919 (0.020)	1.56
Nuclear family	0.517 (0.017)	0.83	0.439 (0.032)	1.56	0.535 (0.031)	1.20	0.500 (0.023)	0.96
Slum household (with tenure)	0.217 (0.452)	2.43	0.172 (0.33)	2.13	0.330 (0.044)	1.83	0.919 (0.023)	1.84
Slum household(without tenure)	0.184 (0.039)	2.39	0.140 (0.031)	2.18	0.275 (0.043)	1.87	0.008 (0.006)	1.38
Urban poverty index	0.320 (0.060)	3.08	0.229 (0.038)	2.21	0.770 (0.032)	1.50	0.040 (0.019)	2.11
Migrant (head of HH)	0.700 (0.056)	2.96	0.780 (0.025)	1.48	0.543 (0.034)	1.32	0.665 (0.070)	3.22
Married	0.675 (0.014)	1.23	0.663 (0.026)	2.13	0.758 (0.017)	1.30	0.723 (0.018)	1.46
Employed full-time	0.459 (0.022)	1.82	0.486 (0.028)	2.21	0.523 (0.019)	1.20	0.584 (0.034)	2.47
Male 18+	0.371 (0.013)	1.34	0.416 (0.022)	2.02	0.319 (0.009)	0.79	0.317 (0.017)	1.52
Secondary+ education	0.495 (0.042)	3.99	0.528 (0.032)	2.95	0.145 (0.014)	1.54	0.568 (0.014)	1.13

#### Sample Frame

We used WorldPop gridded population estimates as sample frames rather than older censuses. At the time of planning, the last censuses in Nepal (2011), Bangladesh (2011), and Vietnam (2009) were seven or more years old [34]. WorldPop is modeled with a machine learning approach that disaggregates UN-adjusted population counts from administrative areas to approximately 100 × 100 m grid cells based on dozens of recently collected spatial covariates derived from satellite imagery and GIS data [35]. This means that total population counts, and the spatial distribution of these populations, are likely more accurate than the last census. The small size of grid cells enables area-microcensus sampling. The Kathmandu sample was drawn from 2017 WorldPop estimates, while the Dhaka and Hanoi surveys were drawn from 2020 WorldPop estimates produced in 2017 and 2013, respectively (Table 1) [34].

#### Sample Selection

At the time of survey, the GridSample R package was the only publicly available tool to perform PPS sampling from gridded population data [36]. The algorithm allows aggregation of population estimates to larger cells (e.g., 200 × 200 m) and selection with PPS. Users can optionally “grow” non-overlapping clusters to a minimum population by randomly adding neighboring cells to selected “seed” cells. This is not ideal, as sampling units should be formed before sampling; however, gridded population sampling tools with this capability were only recently developed [37]. We used the population in the “grown” sampling unit for sample weight calculations following the logic that a frame of “grown” sampling units is implied in the sample weights calculation (Appendix) [36]. Theoretically an adaptive sample weight could be calculated [38]; however, the number of terms required for all combinations of potential cells that could be covered by the “growth” algorithm approaches infinity. In the Kathmandu two-stage sample, households were systematically sampled in Excel following standard methods [13, 14, 39].

#### Cell Size

In Kathmandu, all clusters were initially sampled from 100 × 100 m cells and “grown” to a minimum of 820 people (approximately 200 households) (Table 1). Among these 60 selected clusters, half were randomized to the area-microcensus arm and given the boundary of the original 100 × 100m “seed” cell (Fig. 1). In Dhaka, the sample frame comprised 100 × 100 m cells, and in Hanoi, the sample frame comprised 200 × 200 m cells (Fig. 1). The optimum cell size for each survey was determined using satellite imagery (SUE training manual [39]).

#### Pre-field Review and Segmentation

We visualized each cluster boundary over satellite imagery in ArcGIS before producing field maps and manually segmented clusters that clearly exceeded 200 (two-stage) or 20 (area-microcensus) households. Segment boundaries followed roads and property fences and had approximately equal populations; then, one segment was selected at random to represent the cluster (Fig. 1).

#### Mapping-Listing Protocols

The mapping-listing trainings were each one-week and involved lectures, role-play, group discussion, and a field test. Before fieldwork, mappers-listers updated buildings, roads, and pathways in each cluster in OpenStreetMap using the iD editor tool [40]. In ArcGIS, the survey planning teams used the updated OpenStreetMap layer and cluster boundaries to create a geographically accurate map for each cluster (Fig. 1) [41]. In the field, mappers-listers noted changes on the paper map, followed a script to approach residents, and upon request, distributed a written description of the survey. The household listing was collected in GeoODK, an OpenDataKit-based application [42], for all buildings within the cluster or intersected by its boundary. Mappers-listers commuted from home to assigned nearby clusters using a provided stipend. Daily, they submitted listing records and an image of the field map, and periodically they visited the office to debrief and update OpenStreetMap with changes noted on paper maps.

#### Post-field Segmentation (Area-Microcensus)

To ensure that interviewers would find approximately 20 households in each area-microcensus cluster, any such cluster with more than 25 dwellings was segmented manually in ArcGIS by a GIS specialist and the survey coordinator after mapping-listing fieldwork, ensuring equal numbers of dwellings in each segment [39].

#### Household Definitions

The DHS and MICS define household members as (i) usual residents or people who slept in the dwelling the previous night and who (ii) share living arrangements and (iii) share food [13, 14]. The LSMS defines household members as (i) people who slept in the dwelling three or more of the last 12 months and (ii) share food [15]. By all DHS, MICS, and LSMS definitions, households in both residential and commercial buildings should be included [13–15], guards and servants are subsumed into the household of their employment [13–15], and seasonal and migrant populations are usually excluded by design [43]. The SUE household definition was broader and simply included all self-reported usual residents. The SUE definition additionally included hostel-dwellers and long-term occupants of guesthouses (defined as last 7+ consecutive days and working, looking for work, or in the city for another purpose such as supporting someone in hospital), and street-sleepers who slept in the cluster the previous night. Servants (and their families) who lived at the employer’s residence were counted as a separate household [39].

#### Interview Protocols

In the Kathmandu two-stage arm, geospatial specialists mapped and listed households, while public health specialists conducted interviews with sampled households later (Table 1). In Kathmandu and Dhaka’s area-microcensus samples, geospatial experts mapped and listed *dwellings*, and the household listing was performed by interviewers on the day of interview. Due to time constraints in Hanoi, mapping, listing, and interviews were wrapped into one activity and conducted by public health specialists. This meant that maps used by interviewers in Kathmandu and Dhaka were field-verified, while in Hanoi, maps had only been updated during pre-field enumeration using satellite imagery.

In all three surveys, the SUE household definition was used to determine eligibility, and respondents provided written informed consent and were 18+ years of age and usually a senior household member. The interviewers read questions and recorded responses on a tablet in GeoODK. The household questionnaire collected demographics, assets, income/savings/expenditures, social capital, migration, and injury information. We also collected information about living arrangements, meals, and length of time at the dwelling to classify individuals and households that met DHS/MICS and LSMS definitions during analysis. One adult in each household was randomly selected using the Kish method to complete an individual questionnaire with mental health and migration questions [44].

### Public Involvement

Members of the public, including survey respondents, were not involved in setting the research questions, outcome measures, design, or implementation of the study, nor the dissemination of study results.

### Statistical Evaluation

Sample weights were calculated separately according to the SUE and DHS/MICS household definitions. We analyzed survey results in Stata 14.0 with svy commands, adjusting for sample weights and estimating Taylor-linearized variances to account for clustering of observations within clusters (and household definition in select analyses—see below). The analyses in Kathmandu were stratified by arm (area-microcensus/two-stage), and the analysis in Dhaka was stratified by community (ward/slum).

In the area-microcensus samples in all cities, we evaluated whether use of the DHS/MICS household definition resulted in different estimates of individual and household characteristics compared with use of the SUE household definition using percentages and logit regression at 5% alpha level with “exclusion from DHS/MICS” as the dependent variable and one characteristic as the independent variable. In these comparisons, the DHS/MICS households are a subset of the SUE households and thus treated in regressions as a matched pair by including “SUE vs. DHS/MICS ID” in the svyset statement as a second-stage cluster to correctly estimate variances and differences (*p* values). This approach with dichotomous variables is the survey analysis equivalent of the McNemar test for paired data [45]. In the Kathmandu sample, we also used percentages and logit regression to compare whether characteristics differed in the area-microcensus versus two-stage sample: first, holding the DHS/MICS household definition constant, and second, comparing two-stage-DHS/MICS with area-microcensus-SUE households. Because the households are from independent samples in this comparison, variance estimates (*p* values) adjusted only for the clustering of households within cluster. For every 20 comparisons, we would expect one comparison to be statistically significant by chance (type I error). With this in mind, our interpretation focuses on characteristics which were statistically significant, and for which a large percentage and number of people were excluded.

Household characteristics included building type, member configuration, migration status of household head, slum household, and urban poverty index (UPI) [46]. Individual characteristics included age-gender groups, employment status, marital status, and highest level of education. A reference group was selected for each variable to make statistical comparisons, and observations were dropped if they lacked data to determine household definition eligibility.

Days worked by each staff member and costs were recorded by the survey coordinator in each city. Time spent by survey coordinators to develop and learn the novel methods was excluded from cost calculations. However, time spent training mappers-listers and interviewers was included. In Kathmandu, we estimated costs for the area-microcensus and two-stage arms separately by holding constant costs of administration, training, and durable goods, and varying days of fieldwork.

### Qualitative Evaluation

An FGD was held with each of mapping-listing teams using the same guide covering topics of OpenStreetMap enumeration, mapping-listing, and workflow. Additional questions exploring differences in area-microcensus and two-stage clusters were included in the Kathmandu FGD. FGDs were facilitated and audio-recorded by two trained qualitative researchers and conducted in the local language. The recordings were transcribed into the local language and then translated into English. We performed a thematic Framework Analysis in NVivo 11, coding every line by theme and summarizing positive/neutral experiences, challenges, and recommendations [47].

### Ethics

Ethics approvals were obtained from the University of Leeds (ref.: MREC16-137), University of Southampton (ref.: 26819), Nepal Health Research Council (ref.: 1761), Bangladesh Medical Research Council (ref.: BMRC/NREC/RP/2016-2019/317), and Hanoi University of Public Health (ref.: 324/2017/YTCC-HD3).

## Results

In Kathmandu, 15% of clusters were dropped and replaced. No clusters were dropped in the targeted areas of Dhaka, and 45% were dropped and replaced in the Hanoi district (Table 1). Due to high density in Dhaka, and larger clusters in Hanoi, nearly all clusters in those cities required segmentation to achieve 20 households per cluster (Table 1). Household response rates were 96.8% in the Kathmandu two-stage arm, 88.3% in the Kathmandu area-microcensus arm, 98.7% in Dhaka, and 82.7% in Hanoi (Table 1). The treatment of survey arms as strata in the Kathmandu sample meant that weights were larger in the two-stage arm because clusters comprised larger populations (mean: 1.673, range: 0.298–5.524) than in the area-microcensus arm (mean: 0.347, range: 0.157–0.985) (Table 1). The root design effects (DEFTs) for key demographic and socioeconomic outcomes were larger in area-microcensus units for demographic indicators, but smaller in area-microcensus units for slum household, UPI, migrant status, and education indicators (Table 1).

### Unintentional Exclusion due to Household Definition

Across the area-microcensus samples, applying the DHS/MICS or LSMS household definition resulted in exclusion of approximately 10% of households (unweighted) compared with the SUE definition (Table 1). In Kathmandu, nearly half (46.9%) of single adult households and sizable portions of migrant-headed households (6.7%), non-married (8.5%), unemployed (10.5%), disabled (9.3%), and studying (14.3%) adults were excluded by the DHS/MICS definition (Table 2). In the Dhaka and Hanoi surveys targeting vulnerable communities, sizable portions of single adult households (95.0 and 47.6%), non-married (48.1 and 37.3%), unemployed (32.6 and 23.9%), retired (70.5 and 27.6%), disabled (48.9 and 55.2%), studying adults (81.4 and 84.0%), young people (59.4–79.8% and 88.5–92.7%), and adult women (50.6 and 18.4%) were excluded by the DHS/MICS household definition (Table 2).

**Table 2:** Unintentional exclusion due to household definition: percent of population who would be excluded using the standard DHS/MICS versus SUE household definition in Kathmandu, Dhaka, and Hanoi. All sampled (SUE) households in each area-microcensus sample were split by (a) those who met the DHS/MICS household definition and (b) those who did not. We present the percent of households excluded from the DHS/MICS household definition, and regression coefficient *p* value comparing (a) and (b)

Indicator	Kathmandu Area-microcensus sample only				Dhaka Area-microcensus sample				Hanoi Area-microcensus sample			
	N-wgt SUE (incl. DHS/MICS)	% included by DHS/MICS	% excluded by DHS/ MICS	*p* value^†^	N-wgt SUE (incl. DHS/MICS)	% included by DHS/MICS	% excluded by DHS/ MICS	*p* value^†^	N-wgt SUE (incl. DHS/MICS)	% (N-wgt) included by DHS/MICS	% excluded by DHS/ MICS	*p* value^†^
Households												
Configuration												
Single adult	22	53.1	46.9	< 0.001	24	5.0	95.0	< 0.001	43	52.4	47.6	0.002
One woman with children	10	100.0	0.0	–	9	92.1	7.9	0.967	6	33.3	66.7	0.006
Nuclear family	91	99.4	0.6	Ref.	205	91.7	8.3	Ref.	231	98.6	1.4	Ref.
Other family*	73	99.4	0.6	0.905	143	89.4	10.6	0.579	147	93.0	7.0	0.042
Non-family	13	100.0	0.0	–	1	10.5	89.5	0.013	35	58.8	42.2	0.001
Slum household** (with tenure)												
No	172	94.9	5.1	Ref.	295	84.1	15.9	Ref.	31	82.3	17.7	Ref.
Yes	36	93.4	6.6	0.809	87	88.6	11.4	0.281	425	90.0	10.0	0.485
Missing	0	–	–	–	0	–	–	–	7	27.7	72.3	0.120
Slum household** (without tenure)												
No	179	95.0	5.0	Ref.	318	84.2	15.8	Ref.	456	88.7	11.3	Ref.
Yes	29	92.6	7.4	0.722	64	89.7	10.3	0.341	4	92.6	7.4	0.717
Missing	0	–	–	–	0	–	–	–	3	86.9	31.1	0.109
Urban poverty index												
Non-poor	161	94.8	5.2	Ref.	88	90.3	9.7	Ref.	444	89.1	10.9	Ref.
Poor	48	94.3	5.7	0.930	294	83.5	16.5	0.164	19	76.6	23.4	0.160
Migration status (head)												
Non-migrant	46	99.7	0.3	Ref.	174	89.4	10.6	Ref.	155	90.0	10.0	Ref.
Migrant	162	93.3	6.7	0.016	208	81.5	18.5	0.171	308	87.9	12.1	0.483
Adults 18+												
Marital status												
Not married	184	91.5	8.5	0.001	247	51.9	48.1	< 0.001	331	62.7	37.3	0.001
Married	364	97.7	2.3	Ref.	779	70.4	29.6	Ref.	868	91.4	8.6	Ref.
Missing	0	0	–	–	1	100.0	0.0	–	3	68.0	32.0	0.310
Employment status												
Full-time employed	267	98.4	1.6	Ref.	538	91.7	8.3	Ref.	702	93.1	6.9	Ref.
Part-time, underemployed	10	100.0	0.0	–	37	87.5	12.5	0.556	39	93.0	7.0	0.989
Unemployed	27	89.5	10.5	0.001	46	67.4	32.6	0.003	92	76.1	23.9	0.007
Retired	20	98.1	1.9	0.839	307	29.5	70.5	< 0.001	46	72.4	27.6	0.041
Homemaker	123	98.5	1.5	0.860	2	53.4	46.6	0.133	215	85.6	14.4	0.004
Disabled “unable to work”	17	90.7	9.3	0.009	34	51.1	48.9	0.002	21	44.8	55.2	< 0.001
Student	82	85.7	14.3	0.003	57	18.6	81.4	< 0.001	82	16.0	84.0	< 0.001
Missing	2	0.0	100.0	–	6	24.8	75.2	0.012	5	81.0	19.0	0.448
Individuals												
Gender and age group												
Male < 12	55	98.6	1.4	0.139	206	22.7	77.3	< 0.001	207	10.4	89.6	< 0.001
Female < 12	48	98.4	1.6	0.291	180	20.2	79.8	< 0.001	157	11.5	88.5	< 0.001
Male 12–17	31	95.1	4.9	0.822	105	40.2	59.8	< 0.001	78	7.3	92.7	< 0.001
Female 12–17	32	96.6	3.4	0.442	87	40.6	59.4	< 0.001	47	9.3	90.7	< 0.001
Male 18+	297	94.3	5.7	Ref.	512	82.5	17.5	Ref.	536	85.8	14.2	Ref.
Female 18+	251	97.2	2.8	0.203	514	49.4	50.6	< 0.001	665	81.6	18.4	0.239
Missing	0	0.0	–	–	2	100.0	0.0	–	0	–	–	–
Level of education												
Less than primary	171	95.3	4.7	0.733	906	48.8	51.2	0.062	340	20.2	79.8	< 0.001
Primary	124	95.4	4.6	0.711	353	55.1	44.9	0.803	232	64.0	36.0	0.012
Secondary+	377	96.1	3.9	Ref.	233	56.4	43.6	Ref.	960	84.6	15.4	Ref.
Missing	42	100.0	0.0	–	113	61.9	38.1	0.449	158	14.5	85.5	< 0.001

*N-wgt* weighted count

*Includes living with servants and/or extended family, sometimes with non-family household members as well

**Defined as lacking improved water, improved sanitation, a durable structure, sufficient sleeping space (based on DHS/MICS household member definition), or insecure tenure

^†^Logit regression

### Unintentional Exclusion due to Sample Design

Applying the DHS/MICS household definition, we compared area-microcensus and two-stage samples in Kathmandu to understand how sample design might influence types of respondents (Table 3). We found average household size was smaller in the area-microcensus sample, but dwellings had more occupants (household: 3.5 vs. 3.9, dwelling: 5.0 vs. 3.9) (Table 3). Further, the area-microcensus design had more non-family households (6.0% vs. 1.9%), but the two-stage design included more shack and tent dwellers (0.7% vs. 3.8%) (Table 3).

**Table 3:** Unintentional exclusion due to sample design and household definition: Kathmandu sample characteristics comparing (a) two-stage DHS/MICS versus area-microcensus DHS/MICS and (b) two-stage DHS/MICS versus area-microcensus SUE

Indicators	Two-stage DHS/MICS (Ref.)		Area-microcensus DHS/MICS			Area-microcensus SUE		
	N-wgt	Mean or percent	N-wgt	Mean or percent	*p* value^†^	N-wgt	Mean or percent	*p* value^†^
Survey metrics								
HH size	928	3.9	191	3.5	0.014	208	3.4	0.013
Dwelling size	928	3.9	191	5.0	< 0.001	208	5.3	0.001
HHs per PSU	928	19.5	191	23.4	0.016	208	24.9	0.051
Households								
Building type								
Residential	681	73.4%	137	71.8%	Ref.	142	68.2%	Ref.
Mixed	206	22.2%	50	26.4%	0.595	52	25.0%	0.594
Commercial	6	0.7%	3	1.2%	0.447	2	1.2%	0.450
Shack or tent	35	3.8%	1	0.7%	0.009	1	0.6%	0.009
Hostel	0	–	0	–	–	8	3.8%	< 0.001
Street-sleeper	0	–	0	–	–	2	1.0%	< 0.001
Guesthouse	0	–	0	–	–	0	0.1%	< 0.001
Configuration								
Single adult	42	4.5%	11	5.8%	0.256	22	10.4%	0.040
One woman with children	29	3.2%	10	4.9%	0.093	10	4.7%	0.096
Nuclear family	480	51.7%	88	46.1%	Ref.	91	43.9%	Ref.
Other family*	360	38.8%	70	36.8%	0.600	73	35.1%	0.603
Non-family	17	1.9%	12	6.3%	0.029	13	6.0%	0.030
Slum household** (with tenure)								
No	729	78.5%	158	83.0%	Ref.	172	82.8%	Ref.
Yes	199	21.5%	32	17.0%	0.393	36	17.2%	0.418
Urban poverty index								
Non-poor	633	68.2%	147	77.2%	Ref.	161	77.1%	Ref.
Poor	295	31.8%	44	22.8%	0.189	48	22.9%	0.201
Migrant (head)								
No	280	30.1%	44	23.2%	Ref.	46	22.1%	Ref.
Yes	648	69.9%	147	76.8%	0.244	162	78.0%	0.173
Adults 18+								
Marital status								
Not married	861	32.5%	163	32.2%	0.924	185	33.7%	0.107
Married	1786	67.5%	344	67.8%	Ref.	363	66.3%	Ref.
Employed full-time								
No	1430	54.0%	253	49.9%	0.253	280	51.1%	0.430
Yes	1217	46.0%	254	50.1%	Ref.	267	48.7%	Ref.
Missing	0	–	0	–	–	1	0.3%	< 0.001
Individuals								
Age, gender group								
Male < 12	334	9.4%	52	7.9%	0.149	55	7.7%	0.089
Female < 12	232	6.5%	46	6.7%	0.875	48	6.7%	0.710
Male 12–17	170	4.8%	29	4.3%	0.287	31	4.4%	0.275
Female 12–17	181	5.1%	30	4.5%	0.330	32	4.5%	0.275
Male 18+	1329	37.3%	271	40.8%	Ref.	297	41.6%	Ref.
Female 18+	1318	37.0%	236	35.6%	0.202	251	35.2%	0.118
Education								
Less than primary	957	26.9%	157	23.8%	0.412	171	23.9%	0.440
Primary	599	16.8%	115	17.3%	0.880	124	17.4%	0.906
Secondary+	1774	49.8%	351	52.9%	Ref.	377	52.8%	Ref.
Missing	234	6.6%	41	6.1%	0.601	42	5.9%	0.494

*N-wgt* weighted count

*Includes living with servants and/or extended family, sometimes with non-family household members as well

**Defined as lacking improved water, improved sanitation, a durable structure, sufficient sleeping space, or insecure tenure

^†^Logit regression

### Unintentional Exclusion due to Sample Design and Household Definition

Building off the previous analysis, we compared the area-microcensus sample with SUE definition and the two-stage sample with DHS/MICS definition in Kathmandu to understand the combined effects of survey design and household definition. In the area-microcensus-SUE sample, there were more single adult (10.4% vs. 4.5%) and non-family households (6.0% vs. 1.9%), plus inclusion of hostel-dwellers (3.8%), street-sleepers (1.0%), and long-term guesthouse residents (0.1%) who did not meet the DHS/MICS household definition (Table 3). However, the two-stage-DHS/MICS sample included more shack and tent dwellers (0.6% vs. 3.8%) (Table 3).

### Time and Cost

In Kathmandu, the area-microcensus gridded population survey arm with a target of 600 households in 30 clusters cost approximately US$26,769, or US$45 per household, while a comparable two-stage survey cost approximately US$35,284, or US$59 per household. Area-microcensus survey costs per household in Dhaka (US$34) and Hanoi (US$76) differed due to cost of living and limited economy of scale in those smaller samples. The main cost difference between Kathmandu’s survey arms was the mapping-listing activity; costs were 2.5 times greater in the two-stage arm due to larger clusters (Table 4).

**Table 4:** Comparison of time and budget to perform area-microcensus versus two-stage survey (estimated) in Kathmandu, Dhaka, and Hanoi

Budget item	Kathmandu, two-stage		Kathmandu, area-microcensus		Dhaka, area-microcensus		Hanoi, area-microcensus	
	Time	Cost USD	Time	Cost USD	Time	Cost USD	Time	Cost USD
Planning and administration	75 days		60 days		60 days		20 days	
Salaries		9240		8006		4305		7468
Mapping-dwelling/HH listing-GIS	35 days × 6 mapper-listers 1 GIS specialist		12 days × 6 mapper-listers 1 GIS specialist		36 days × 8 mapper-listers 1 GIS specialist		8 days × 12 listers	
Salaries, per diem		7641		3056		4926		6128
Materials		291		218		120		68
Interviews and data management	19 days × 8 interviewers		15 days × 8 interviewers		24 days × 7 interviewers		13 days × 12 interviewers	
Salaries, per diem		5723		4518		2345		11,872
Materials, including pilot		2106		2106		872		574
Incentives, local collaborators		0		0		0		3089
Ethics review		1998		1998		238		1362
Equipment								
Laptops/hard drives		1193		1193		167		0
Tablets		1212		1212		382		1714
Overhead	20% direct costs	5786	20% direct costs	4367	20% direct costs	2671	10% direct costs	3228
Total		35,284		26,769		16,026		35,503
Per household		59		45		34		76

### Skill Mix

The skills required to plan and implement SUE surveys were similar to standard household surveys. The main difference was skillset of the mapping-listing team. In a standard survey, mapping-listing staff are required to have a secondary education [48]. To use SUE tools and methods, the mapping-listing staff should additionally have training in geography, GIS, or related fieldwork and be comfortable using mobile technologies for data collection and navigation. The skillsets of other staff including survey planners, trainers, and interviewers were identical to a standard household survey. The GridSample R package required intermediate R programming and GIS skills; however, a free point-and-click tool called gridsample.org is now available, allowing non-technical design and implementation of gridded population surveys.

### Experiences

Feedback from the mapper-lister FGDs was generally neutral or positive, and staff resoundingly said they would prefer SUE tools and protocols to a conventional paper-based protocol. The SUE survey fieldwork, however, was not without limitations.

#### Key Challenges

In Kathmandu, the mapping-listing staff were comprised of university geospatial students. Several described approaching residents as their greatest challenge, as well as their greatest reward. One mapper-lister explained, “It was fun to work at the social level and interacting with the local people. We always used to be limited to using the computers before.” Mappers-listers added that role-play and practical activities prepared them for fieldwork, though additional training on the survey aims would have helped to explain the survey’s purpose to residents. In Kathmandu, mapping-listing staff initially enumerate 20–30 households daily, and this increased to 40–50 households daily after a week.

The challenges in Dhaka and Hanoi were different. In these cities, the survey planners were trained about SUE tools and protocols but did not have field experience before training mapper-listers and interviewers. As a result, mapping-listing staff, including the geospatial students in Dhaka, described challenges using the tablet applications during the first days of fieldwork. In Hanoi where public health experts performed mapping, listing, and interviews, staff additionally struggled with navigation. Due to community skepticism following recent government evictions in Hanoi, teams enlisted local guides to help approach residents and introduce the survey.

Across cities, mappers-listers described working in pairs as essential because it provided them with “mutual support” to adapt to the moods and reactions of residents, interact in more languages, and work faster with more accuracy. Overwhelmingly, mappers-listers recommend that teams be comprised of one geospatial and one public health specialist.

#### Response Rates

In all three cities, mapping-listing staff reported that residents seemed to omit mention of neighbors who did not have official mortgages or rental contracts, presumably for fear of evictions or fines. This was a particular challenge in Hanoi where “people tended to answer our question following their household record book,” an official registry of households administered by the government. One mapper-lister-interviewer explained, “for residents who were living in evacuated houses, they felt worry and scare as if something wrong could happen.”

In Hanoi, teams returned to each cluster multiple times to build trust with residents and identify households not reported during previous visits. While the presence of guides likely improved response rates, it also meant that survey teams were limited by guides’ schedules. Most teams performed the listing and interviews in the evenings when guides were home, though this meant that residents were eating dinner and rushed, or refused. Mapper-listers and interviewer in Kathmandu and Dhaka performed their work during the day.

Residential building access was a problem across cities. The Hanoi teams faced secured apartment buildings without a guard. In these situations, the planning team contacted the building management boards and were usually able to gain access to these buildings; however once inside, mappers-listers-interviewers often found that residents knew little about their absent neighbors. Kathmandu had wealthy “VIP” neighborhoods and mapping-listing staff reported substantial skepticism and non-response in these neighborhoods.

#### Travel

Mapping-listing staff commuted to clusters via bus, rickshaw, motorbike, and foot. In Kathmandu, most staff never traveled more than 1 h to a cluster; however, a team working in peri-urban Kathmandu spent 3 h commuting one way to one cluster due to the absence of buses or taxis. In Dhaka, where traffic is notoriously bad, commute times to clusters ranged from 1.5 to 3 h. Across the three cities, mapping-listing staff recommended hired vehicles to save time.

#### Area-Microcensus versus Two-Stage Clusters

Mappers-listers in Kathmandu reported different experiences in area-microcensus and two-stage clusters. The two-stage clusters were, by definition, ten times the size of area-microcensus clusters resulting in extra days of work and more physical barriers to navigate such as hills and rivers. In addition, the two-stage clusters required more information than area-microcensus clusters, resulting in longer interactions and higher levels of skepticism among residents.

Residents in Kathmandu were generally willing to report the number of apartments/dwellings per building; however, they were reluctant to specify the number of households per dwelling and to give household head names. In many two-stage clusters, teams approached a business owner on the ground level who gave number of dwellings on the above floors, but refused to give household-level information, and instead directed the mapping-listing staff to the building owner. One way that mappers-listers addressed this challenge was to approach people at a local grocery store and start a conversation away from their building. In this context, residents were less likely to feel they were speaking on behalf of the landlord.

#### Technology

Across sites, mapping-listing staff faced challenges with the tablet applications. While some challenges could have been averted with more, or better, training, other challenges were inherent to the tools and protocols used. First, although OpenStreetMap was updated by mappers-listers before visiting clusters, the updates in various applications occurred on different schedules resulting in different versions of the same map in the field. Specifically, updates to ArcGIS (from which field maps were printed), GeoODK (to collect building GPS points during the listing), and OSMAnd and MAPS.ME (used for navigation) were updated 1–30 days after a change was made to OpenStreetMap.

A second problem was the number of unintegrated applications that the mapping-listing staff were expected to use, resulting in lost time and confusion. Despite having multiple navigation applications and a paper map, mappers-listers in all cities reported delays and difficulty navigating to clusters. Once in a cluster, however, mappers-listers did not report challenges identifying cluster boundaries, despite their blocky shapes. Mappers-listers also found recording the listing data in GeoODK was arduous, and they often took notes on paper when speaking to residents and then entered information into the tablet immediately after.

Third, the location precision within OSMAnd and GeoODK was poor, often showing a circle up to 36 m in which the tablet could be located. Location precision was a particular problem in high-density areas (presumably with tall buildings blocking or refracting signals) and resulted in a few instances of a mapping-listing team starting their work and then realizing that they were recording data one or two streets away from the cluster.

## Discussion

By comparing DHS/MICS and SUE household definitions, and area-microcensus and two-stage sampling, we found evidence that standard household survey methods unintentionally omit single adults and non-family households, both of which are more likely to represent disjoined households or be mobile compared with stable nuclear family households [17, 43, 49]. This is among the first studies in a LMIC context to evaluate under-coverage due to survey design and methods in face-to-face surveys; such studies tend to be conducted in high-income countries [18, 50].

Although the same protocols and household definitions were used to identify households in Kathmandu’s area-microcensus and two-stage arms, the quality of the household listing data appeared to be more thorough in area-microcensus clusters where interviewers (rather than mapper-listers) listed households. Interviewers had more skills to interact with the public and substantially more time at each building while administering questionnaires (2.5–3 h per household as opposed to 15 min per household) to build rapport with residents and learn about atypical and informal housing arrangements. Indicator design effects point to another possible benefit of the area-microcensus design. Although one might expect larger design effects in area-microcensus clusters because near neighbors are assumed to be more similar than far neighbors [31], the DEFTs for slum, migration, and education indicators in area-microcensus clusters were smaller than in two-stage clusters. This might indicate better coverage of the heterogeneous mix of urban residents and better identification of atypical and “hidden” households. Smaller design effects for similar indicators (less than primary education, willingness to take risks, and mental health status) were consistent with a similar study comparing area-microcensus with standard probability sampling in a South African city [32]. Others argue that standard household definitions are no longer suitable in complex LMIC cities; rather, individuals and communities are more appropriate units of measurement [5, 49]. Further research is needed to evaluate potential trade-offs and benefits of moving the household listing responsibility to interviewers using area-microcensus survey designs, but our findings suggest multiple benefits.

Without urban strata, the two-stage sample in Kathmandu was better able to measure tent and shack dwellers than the area-microcensus sample, likely due to the larger area of two-stage clusters. The only way to ensure representative surveys of shack/tent dwellers and other vulnerable populations concentrated in slums is to treat deprived/non-deprived areas as strata, in both area-microcensus and two-stage designs. Others have suggested that censuses classify EAs as slum/non-slum to support stratified urban surveys and numerous initiatives to improve the well-being of slum dwellers and the health of cities [20]. Given the resource constraints facing LMICs, adapting methodologies to leverage slum-classified census EA units within existing global programs for household surveys, such as the DHS, would provide greater value for money. Though this approach would only work for censuses that enumerate residents of slums and informal settlements [9]. While stratifying urban populations by slum and non-slum areas would not diminish the need for high-quality informal settlement-specific data such as those generated through the Nairobi Urban Demographic and Health Surveillance System [25], it would fill the gap in the current evidence base for datasets that measure intra- and inter-urban inequities, and allow valid comparison of rural, urban slum, and urban non-slum populations.

We found that response rates in area-microcensus clusters were lower than in two-stage clusters. This may have been due to the greater proportion of vulnerable and mobile households identified in area-microcensus clusters if they were less willing to participate, more likely absent, or felt disempowered to respond. Readers who are interested in area-microcensus survey designs should take account of lower response rates and potentially higher design effects for certain indicators when calculating sample size. The surveys conducted in Dhaka and Hanoi focused on vulnerable and mobile communities, so rates of exclusion identified in this study may have been higher than in the general population.

Societal changes, particularly rapid urbanization in LMICs, have likely caused decay in survey data accuracy due to increased complexity in living arrangements, urban disparity, and population mobility. Not only are vulnerable and mobile populations more likely to be intentionally excluded from surveys, but also they are at increased risk of unintentional, unmeasured exclusion, and their data are masked in urban averages when they are sampled. Given the importance of household survey data to policy-making, planning, and monitoring progress toward development goals, it is time to evaluate new survey tools and protocols that ensure inclusion of all households.

## Electronic Supplementary Material

ESM 1 (DOCX 31 kb)

## Data Availability

De-identified participant data and a data dictionary defining each field are available upon request with ethics approval. Please submit requests to Joseph Paul Hicks (J.P.Hicks@leeds.ac.uk) and Helen Elsey (helen.elsey@york.ac.uk).

## Abbreviation

DHS: Demographic and Health Surveys
EA: Enumeration area
FGD: Focus group discussion
LSMS: Living Standard Measurement Surveys
LMIC: Low- and middle-income country
MICS: Multiple Indicator Cluster Surveys
PPS: Probability proportional to (population) size
SUE: Surveys for Urban Equity
