# Supplementary material for: Addressing Unintentional Exclusion of Vulnerable and Mobile Households in Traditional Surveys in Kathmandu, Dhaka, and Hanoi: a Mixed-Methods Feasibility Study
Source: J Urban Health. 2020 Oct 27;98(1):111–29. doi: 10.1007/s11524-020-00485-z (PMC7873174; doi:10.1007/s11524-020-00485-z)
Supplement: Supplementary file 1 — (DOCX 31 kb) [file 11524_2020_485_MOESM1_ESM.docx]

## Appendix to “Feasibility of innovative tools and methods to improve household surveys in complex urban settings: Multiple methods analysis of the Surveys for Urban Equity (SUE) study in Kathmandu, Dhaka, and Hanoi” by Dana R. Thomson, Radheshyam Bhattarai, Sudeepa Khanal, Shraddha Manandhar, Rajeev Dhungel, Subash Gajurel, et al.

**Sample weight calculations from WorldPop gridded population sample frames.**

Raw household sample weights were calculated using the following formula.

$$w_{hh.raw}=\frac{G_{k}/g_{ik}}{n_{k}} \times\frac{M_{ik}}{m_{ik}} \times\left( b1_{ik}\times b2_{ik} \right) \times\frac{n_{k}}{n_{k*}} \times\frac{m_{k}}{m_{k*}}$$

Where:

$G_{k}$ estimated population in stratum $k$ from WorldPop

$g_{ik}$ estimated population in cluster $i$ in stratum $k$ from WorldPop

$n_{k}$ number of clusters sampled in stratum $k$

$n_{k*}$ number of clusters sampled and visited in stratum $k$

$M_{ik}$ number of households listed in cluster$i$ in stratum $k$

$m_{ik}$ number of households sampled in cluster$i$ in stratum $k$

$m_{k}$ number of households sampled in stratum $k$

$m_{k*}$ number of households sampled with a completed interview in stratum $k$

$b1_{ik}$ number of pre-field segments in cluster$i$ in stratum $k$

$b2_{ik}$ number of post-field segments in cluster$i$ in stratum $k$ (area-microcensus)

Raw individual sample weights were calculated using the following formula with each raw household sample weight.

$$w_{ind.raw}=w_{hh.raw} \times\frac{U_{ik}}{u_{ik}} \times\frac{u_{k}}{u_{k*}}$$

Where:

$U_{ik}$ number of eligible adults listed in cluster$i$ in stratum $k$

$u_{ik}$ number of eligible adults sampled in cluster$i$ in stratum $k$

$u_{k}$ number of eligible adults sampled in stratum $k$

$u_{k*}$ number of eligible adults sampled with a completed interview in stratum $k$

Household and individual raw weights were normalised by applying the following formulas:

$$w_{hh.norm}=w_{hh.raw} \times\frac{\sum(m_{ik*})}{\sum(w_{hh.raw} \times m_{ik*})}$$

$$w_{ind.norm}=w_{ind.raw} \times\frac{\sum(u_{ik*})}{\sum(w_{ind.raw} \times u_{ik*})}$$

Where:

$w_{hh.raw}$ raw household sample weight in cluster $i$ in stratum $k$

$m_{ik*}$ number of households sampled with completed interview in cluster $i$ in stratum $k$

$w_{ind.raw}$ raw individual sample weight in cluster $i$ in stratum $k$

$u_{ik*}$ number of adults sampled with a completed interview in cluster $i$ in stratum $k$
